# Reliability of goniometric measurements in children with cerebral palsy: A comparative analysis of universal goniometer and electronic inclinometer. A pilot study

**DOI:** 10.1186/1471-2474-12-155

**Published:** 2011-07-10

**Authors:** Pablo Herrero, Patricia Carrera, Elena García, Eva M Gómez-Trullén, Bárbara Oliván-Blázquez

**Affiliations:** 1 Faculty of Health Sciences, San Jorge University, Autovía A 23 Zaragoza-Huesca, km 510, 50830 Villanueva de Gállego (Zaragoza), Spain; 2 AIDIMO, Belle Epoque n° 27, 50019 Zaragoza, Spain; 3 Tecnodiscap Group, University of Zaragoza, c/Maria de Luna n° 1, 50018 Zaragoza, Spain; 4 Aragonese Institute of Health Sciences, Avda. Gómez Laguna n° 25, 50009 Zaragoza, Spain; 5 Department of Physiatry and Nursing, University of Zaragoza, c/Domingo Miral s/n 50009 Zaragoza, Spain

## Abstract

**Background:**

Even though technological progress has provided us with more and more sophisticated equipment for making goniometric measurements, the most commonly used clinical tools are still the universal goniometer and, to a lesser extent, the inclinometer. There is, however, no published study so far that uses an inclinometer for measurements in children with cerebral palsy (CP). The objective of this study was two-fold: to independently assess the intra and inter-examiner reliability for measuring the hip abduction range of motion in children with CP using two different instruments, the universal two-axis goniometer and electronic inclinometer. A pool of 5 examiners with different levels of experience as paediatric physiotherapists participated. The study did not compare both instruments because the measurement procedure and the hip position were different for each.

**Methods:**

A prospective, observational study of goniometery was carried out with 14 lower extremities of 7 children with spastic CP. The inclinometer study was carried out with 8 lower extremities of 4 children with spastic CP. This study was divided into two independent parts: a study of the reliability of the hip abduction range of motion measured with a universal goniometer (hip at 0° flexion) and with an electronic inclinometer (hip at 90° flexion). The Intraclass Correlation Coefficient (ICC) was calculated to analyse intra and inter-examiner agreement for each instrument.

**Results:**

For the goniometer, the intra-examiner reliability was excellent (>0.80), while the inter-examiner reliability was low (0.375 and 0.475). For the inclinometer, both the intra-examiner (0.850-0.975) and inter-examiner reliability were excellent (0.965 and 0.979).

**Conclusions:**

The inter-examiner reliability for goniometric measurement of hip abduction in children with CP was low, in keeping with other results found in previous publications. The inclinometer has proved to be a highly reliable tool for measuring the hip abduction range of motion in children with CP, which opens up new possibilities in this field, despite having some measurement limitations.

## Background

**Figure 1:**
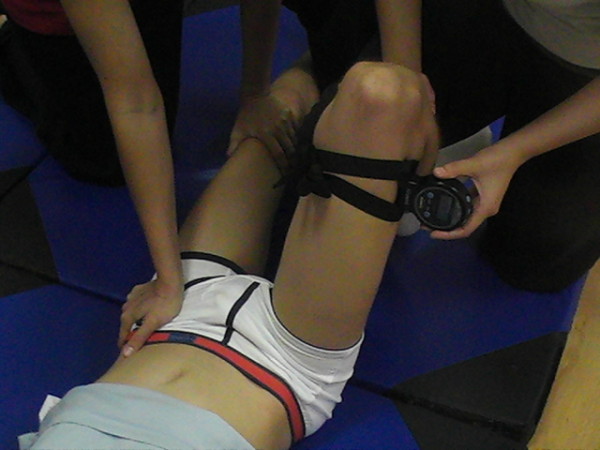
**Inclinometer measurement**. The lower extremity to be measured was held in 90-degree hip flexion, with the contralateral leg stabilised at maximum hip extension by an assistant. The measurement entailed placing the device on the lateral surface of the distal third of the thigh, aligned with the longitudinal axis of the femur.

Goniometric measurements are often used by physiotherapists and physicians to assess muscular shortening and joint stiffness in children with cerebral palsy (CP). The results obtained in the assessment are used to plan rehabilitation treatments and to take decisions regarding the appropriate timing of medical procedures such as serial casting, botulinum toxin infiltration [1-3] and orthopaedic surgery [4-6]. Even though technological progress has provided us with a greater range of sophisticated equipment for making goniometric measurements, the most commonly used clinical tools are still the universal goniometer and, to a lesser extent, the inclinometer (Figure 1). As there is no published evidence of inclinometer for measurements in children with CP, evidence of the reliability of these instruments in children with CP is required in this population with special characteristics.

The studies carried out so far have shown that the number of examiners [7-12] and the particular characteristics of each joint [13,14], among other factors, affect the consistency of the repeated goniometric measurements. Other factors preventing agreement occur when intra-examiner and inter-examiner reliability is studied for goniometric measurements in children with CP. These important determining factors mentioned by different authors include the examiner's experience in differentiating the end range of joint movement due to structural changes and that due to hypertonicity or volitional resistance [15], or the variations in muscle tone of children with CP [15-19].

Nevertheless, the published studies agree that measurements taken by the same examiner within the same session and on the same day have proved to be more reliable than those taken by different examiners between sessions and on different days [15-20]. It has also been observed that this inter-examiner reliability improves with practice [16,17], and that reliability is higher in patients with normal tone than those with hypertonicity [1,3,6,12,15-18,21-27].

Clinically, different authors report measurement errors between 10° and 15° [15,17,18] in goniometric measurements of one-joint muscles in the same session by the same examiner, while this error exceeds 15° in the case of inter-examiner measurements between sessions [15]. Consequently, some authors hold that one must exert caution before basing clinical judgements upon goniometric measurements [15].

The objective of this study was to assess intra and inter-examiner reliability for measuring the hip abduction range of motion in children with CP with two different instruments (the universal two-axis goniometer and electronic inclinometer). A pool of 5 examiners with different levels of experience as paediatric physiotherapists participated. The study did not compare both instruments because measurement procedure and hip position were different for each instrument. Five examiners participated instead of the usual 2 or 3 examiners found in previous studies for two reasons:

1. In Spain and possibly other countries, children with CP are usually assessed by more than 3 health professionals who share patient information before making any decisions.

2. This is a pilot study whose results will be used as a reference to analyze whether the measurements carried out in a financed hippotherapy clinical trial will be reliable when measuring the hip abduction range of motion in 38 children with CP, with different examiners participating and at 0° (goniometer) and 90° of hip flexion (inclinometer).

## Methods

This study funded by the Government of Aragon, was approved by the Aragon Ethics Committee as a preliminary study integrated in a randomised clinical trial aimed at studying the therapeutic benefit of a hippotherapy simulator in children with CP.

Informed consent was given by parents (Current Controlled Trials ISRCTN03663478).

### Participants

The goniometer study included a total of 14 lower extremities of 7 children with spastic CP while the inclinometer one included 8 lower extremities of 4 of the above children with spastic CP (Table 1). The inclinometer study had only 4 children because 3 of the 7 children included in the goniometer study did not attend for the inclinometer study.

**Table 1:** Gender, age and GMFCS level of children with CP included in the sample.

Child	Gender	Age (Years, months)	GMFCS Level
1	Male	11, 1	III

2	Male	15, 2	IV

3	Male	12, 6	IV

4	Male	7, 0	III

5	Male	13, 6	IV

6	Female	7, 6	III

7	Female	15, 0	III

The inclusion criteria were: a) a diagnose of CP and b) spasticity in the hip adductor musculature.

The exclusion criteria were: a) a hip operation during the previous 12 months and b) injection with botulinum toxin during the previous 6 months.

### Study design

This study was divided in two independent, prospective, observational studies: the reliability of the hip abduction range of motion measured with a universal goniometer (hip at 0°) and with an electronic inclinometer (hip at 90° flexion).

Goniometer study: each child was measured in a random order by 5 physiotherapists (Table 2) in the same session to study inter-examiner reliability for the first measurement, and again a week later to study inter-examiner reliability for the second measurement and intra-examiner reliability between the sessions. The two lower extremities of each child were measured one after the other by each examiner. An assistant helped the examiner to stabilise the contralateral lower limb in all measurements, while the rest of the examiners waited outside the consulting room. All examiners were therefore unaware of each other's assessments and agreed to not speak about their results.

**Table 2:** Examination order.

Examiner	Child1*	Child 2*	Child 3	Child 4*	Child 5	Child 6	Child 7
1	1st	2nd	4th	5th	3rd	5th	4th

2	3rd	4th	5th	3rd	2nd	2nd	1st

3	5th	1st	1st	2nd	1st	3rd	3rd

4	4th	5th	3rd	1st	5th	4th	2nd

5	2nd	3rd	2nd	4th	4th	1st	5th

The order in which every child had to be measured by the examiners was randomised. Three (*) of the seven children were not measured in the second study.

Inclinometer study: this was carried out in exactly the same manner as the goniometer study but with the hip position supported at 90° of flexion.

### Examiners

Five examiners with different levels of experience as paediatric physiotherapists (from 1 to 8 years) participated (Table 3). They followed a measurement protocol, which was explained to all of them beforehand and practised by them before using it on the children with CP. A main researcher supervised the measurements to ensure physiotherapists complied with the protocol.

**Table 3:** Gender, age and years of experience of the examiners as paediatric physiotherapists.

Examiner	Gender	Age (years)	Years of experience as a paediatric physiotherapist
1	Female	23	2

2	Male	22	1

3	Female	33	6

4	Female	27	4

5	Male	30	8

### Testing procedure

The method described by Mutlu et al [29] and Stuberg et al [15] was used for the goniometer study. The patient was laid in a supine position, with both legs as parallel as possible, in 0-degree hip flexion-extension and the knees extended. The contralateral leg was stabilised by an assistant to prevent compensatory movements. For the inclinometer study, the lower extremity to be measured was held in 90-degree hip flexion, with the contralateral leg stabilised in maximum hip extension.

The goniometer fulcrum was placed over the anterior superior iliac spine (ASIS) of the hip to be assessed. The stationary arm was directed towards the contralateral ASIS, and the moveable arm aligned with the femur towards the centre of the patella. The initial position of the hip assessed was in 0° abduction, and from this point the range of movement of both hips, for each patient, was measured. The passive abduction movement was performed gently to prevent myotatic reflex, ensuring that the hip being assessed did not rotate (by keeping the orientation of the tip of the toes in the neutral position) and that the pelvis did not tilt. Once the passive movement limit was found, the goniometer was replaced, aligned with the aforementioned bone structures. For the inclinometer, the measurement entailed placing the device on the lateral surface of the distal third of the thigh, aligned with the longitudinal axis of the femur (Figure 1).

**Figure 2:**
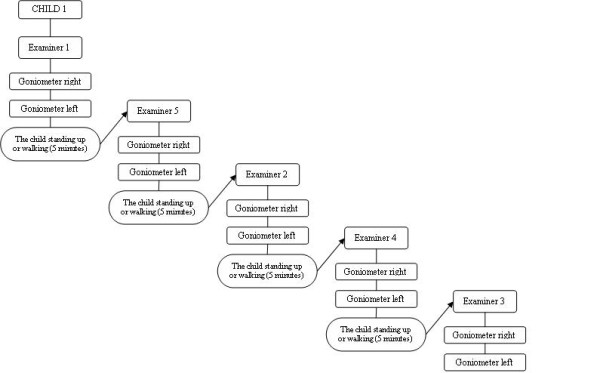
**Goniometer measurement sequence**. Goniometer measurement sequence for child 1. The same procedure was used for the inclinometer measurements.

In both studies, each examiner carried out one measurement for each lower limb. After each examiner had finished, the child stood up or walked around with the help of the assistant for 5 minutes (Figure 2). This was to prevent repeated stretching from interfering with examiners' measurements [18].

The main parameters checked were those regarding standard positioning, stabilisation and identification of the bone structure landmarks, even though there are conflicting opinions regarding whether examiners should be specifically trained or not [15,16,18-20,28]. Examiners were trained to differentiate between the firm limit caused by structural or joint restriction (true end range of motion) and the soft limit caused by volitional resistance or hypertonicity. Prior to performing the measurement on study subjects they practised together and agreed the criteria for identifying the end range of motion.

The stretching time used by each examiner was not standardised, as it was essential that each examiner was sure that he had reached the limit of range. Examiners were trained to move gently until they could feel the joint end-feel, but strength of force applied by the examiners was not measured.

A physiotherapist not involved in the study acted as an assistant collecting data, helping the examiner when needed and ensuring that the process was completely impartial; simulating the daily conditions of a physiotherapist's clinic. Each physiotherapist was assisted by the same assistant physiotherapist, who did not perform any measurement. Although several studies have reported using an assistant to improve the reliability of the measurements [16,17,19,20,28], there is no current evidence available to support the use of an assistant for this purpose.

### Statistical Analysis

An initial descriptive analysis was carried out by calculating measurements of central tendency and distribution as well as 95% confidence intervals in all cases. The Intraclass Correlation Coefficient, using a two factor mixed effects model, was calculated to analyse intra and inter-examiner concordance of each measurement tool. The Student's t test and Pearson's correlation coefficient to prove the relationship between different measurements were also calculated. The distribution of the variables was analysed with the Kolmogorov-Smirnov test. The level of significance assumed was p < 0.05. The statistical package used was SPSS 14.0.

As stated by Mutlu [29] et al, ICCs can vary from 0.00 to 1.00, where values from 0.6 to 0.8 are regarded as evidence of good reliability and those above indicating excellent reliability [8,15,16,18,30-34].

## Results

### Goniometer

The values obtained in the goniometer measures are shown in Table 4. The average of the measurements taken in the two sessions by different examiners was calculated to investigate any significant statistical difference between them.

**Table 4:** Measures of central tendency of the data obtained with the goniometer by the 5 examiners in the first and the second measurement (a week later).

Measurement	Examiner	Mean	CI 95%	Median	**Min**.	**Max**.	K-S (p)
1	1	5.50	3.61-7.39	5	0	10	0.120
	
	2	10.43	8.25-12.61	10	2	15	0.027
	
	3	4.00	2.76-5.24	4	0	8	0.200
	
	4	4.79	2.65-2.92	3.50	1	12	0.049
	
	5	1.82	1.13-2.52	2	0	4	0.127

2	1	5.93	3.99-7.87	6.50	-1	10	0.041
	
	2	10.71	8.11-13.31	10	2	20	0.200
	
	3	3.86	1.87-5.84	4	0	12	0.200
	
	4	4.21	2.20-6.23	4	0	10	0.030
	
	5	1.68	0.90-2.46	2	-1	4	0.032

Normality tests of the measures taken by each examiner.

*K-S (Kolmogorov-Smirnov): normality test. We consider that normality should not be rejected if p > 0.05.

As shown in Table 5, there were no significant differences in measurements between the different sessions, as the Student's t test was not significant in any case. In fact, a high correlation between both measurements taken by each examiner was observed (Pearson's Correlation Coefficient), the concordance was statistically significant (p < 0.05), with the highest ICC for examiner number 4 (ICC = 0.954) and the lowest for number 3 (ICC = 0.828).

**Table 5:** Intra-examiner reliability in the measurement of hip abduction with the universal two-axis goniometer by five examiners.

Examiner	r	ICC1	CI 95%	P	Mean	t	p
1	0.779	0.879	0.630-0.971	<0.001	-0.43	-0.726	0.481

2	0.705	0.829	0.459-0.946	0.002	-0.29	-0.329	0.747

3	0.771	0.828	0.453-0.945	0.002	0.14	0.238	0.816

4	0.920	0.954	0.859-0.985	<0.001	0.57	1.472	0.165

5	0.884	0.936	0.806-0.979	<0.001	0.14	0.844	0.414

r: Pearson's correlation coefficient; CI: Confidence interval; ICC1: Intraclass Correlation Coefficient; t: Student's t test.

When comparing different examiners (Table 6), the agreement was also significant, with an ICC of 0.375 for the first measurement and 0.475 for the second.

**Table 6:** Inter-examiner reliability for measurements 1 and 2 (a week later) of hip abduction with the universal two-axis goniometer.

Measurement	Examiners	r	ICC2	CI 95%	p	T	P
	1-2	0.398				-3.69	0.001
					
	1-3	0.612				1.43	0.164
					
	1-4	0.593				0.54	0.593
					
	1-5	0.259				3.94	0.001
					
	2-3	0.180				5.54	<0.001
1			0.375	-0.008-0.716	0.012		
	2-4	-0.218				3.99	<0.001
					
	2-5	-0.253				8.13	<0.001
					
	3-4	0.464				-0.69	0.500
					
	3-5	0.238				3.31	0.003
					
	4-5	0.198				2.85	0.012

	1-2	0.314					
						
	1-3	0.491					
						
	1-4	0.729					
						
	1-5	0.088					
						
	2-3	0.515					
2			0.475	0.076-0.777	0.002		
	2-4	0.141					
						
	2-5	-0.320					
						
	3-4	0.650					
						
	3-5	-0.209					
						
	4-5	-0.025					

r: Pearson's correlation coefficient; CI: confidence interval; ICC2: Interclass Correlation coefficient; t: Student's t test

### Inclinometer

The same data are shown for the measurements carried out with the inclinometer (Table 7), which show a high degree of intra-examiner correlation, with statistically significant differences only in the reliability of measurements performed by examiner number 2 (Table 8). The inter-examiner correlation coefficient exceeds 0.9 in the two measurements performed (Table 9).

**Table 7:** Values of central tendency of the measures taken with an inclinometer by the 5 examiners in the first and second measure (a week later).

Measurement	Examiner	Mean	CI 95%	Median	**Min**.	**Max**.	K-S (p)
1	1	41.50	32.16-50.84	38.50	26	61	0.859
	
	2	33.63	22.10-45.15	30.00	17	57	0.481
	
	3	33.00	18.82-47.18	28.50	17	63	0.385
	
	4	32.25	16.74-47.76	22.50	19	63	0.311
	
	5	36.75	24.55-48.95	31.50	19	60	0.602

2	1	37.88	28.38-47.37	34.00	23	54	0.813
	
	2	42.63	31.71-53.54	38.50	28	63	0.753
	
	3	36.13	25.08-47.17	31.00	20	57	0.410
	
	4	36.13	23.27-48.98	36.00	16	58	0.993
	
	5	33.38	17.66-49.09	25.00	11	65	0.569

Normality tests of the measurements performed by each examiner.

*K-S (Kolmogorov-Smirnov): normality test. We consider that normality should not be rejected if p > 0.05.

**Table 8:** Intra-examiner reliability in the measurement of hip abduction performed by 5 examiners with an inclinometer.

Examiner	r	ICC1	CI 95%	p	Median	T	p
1	0.739	0.850	0.250-0.970	0.011	3.625	1.260	0.248

2	0.971	0.984	0.922-0.997	<0.001	-9.000	-7.626	<0.001

3	0.961	0.965	0.824-0.993	<0.001	-3.125	-1.574	0.160

4	0.820	0.892	0.462-0.978	0.004	-3.875	-1.031	0.337

5	0.983	0.975	0.877-0.995	<0.001	3.375	1.833	0.109

r: Pearson's correlation coefficient; CI: Confidence interval; ICC1: Intraclass Correlation Coefficient; t: Student's t test.

**Table 9:** Inter-examiner reliability for measurements 1 and 2 (a week later) of hip abduction with an inclinometer.

Measurement	Examiner	r	ICC2	CI 95%	p	t	P
	1-2	0.867				1.255	0.230
					
	1-3	0.928				1.184	0.256
					
	1-4	0.871				1.208	0.247
					
	1-5	0.876				0.731	0.477
					
	2-3	0.950				0.081	0.937
1			0.979	0.940-0.995	<0.001		
	2-4	0.946				0.168	0.869
					
	2-5	0.991				-0.440	0.666
					
	3-4	0.943				0.084	0.934
					
	3-5	0.971				-0.474	0.643
					
	4-5	0.950				-0.539	0.598

	1-2	0.888					
						
	1-3	0.826					
						
	1-4	0.882					
						
	1-5	0.849					
						
	2-3	0.861					
2			0.965	0.903-0.992	<0.001		
	2-4	0.842					
						
	2-5	0.975					
						
	3-4	0.926					
						
	3-5	0.860					
						
	4-5	0.874					

r: Pearson's correlation coefficient; CI: Confidence interval; ICC2: Intraclass Correlation Coefficient; t: Student's t test.

## Discussion

For the goniometer, intra-examiner reliability was excellent (>0.80), while the inter-examiner reliability was low (0.375 and 0.475). These data concur with most of the publications [15-20] although a study by Mutlu et al [29] concluded that inter-examiner reliability was higher than intra-examiner for the hip abduction. However, this was not the generalisable conclusion as reliability varied in other movements, such as hip extension and external rotation. On the other hand, most recent studies published consider inter-examiner reliability of goniometric measurements to be low [15-17,20,28], which coincides with the results obtained in this study. We agree with Mutlu [29], who identified the previous training of examiners as an important factor in inter-examiner reliability, especially concerning the ability to accurately differentiate and determine the true end range, which is sometimes difficult in children with CP. This aspect was especially considered during the study design by carrying out previous training with the examiners. Despite these steps and the fact that the measuring order was randomised for each patient, we think that the low ICC obtained could be due to different factors: (1) the difficulty in fixing the pelvis, preventing compensatory movements, and keeping the stationary and moveable arms correctly aligned with the anatomic references during the whole measuring process; (2) the professional ability of examiners, and not just their experience; (3) having a higher number of examiners.

Some other aspects, such as stretching time, were not standardised, due to the differences in the subjects' active resistance, the ability to relax and other factors, as stated by McWhirk et al. However, we think these aspects had little influence on inter-examiner reliability, as we consider that the physiotherapist's subjective assessment is more reliable than standardising the stretching time.

Regarding measurements with an inclinometer, reliability results for both the intra-examiner (0.850-0.975) and inter-examiner (0.965 and 0.979) were excellent. No study regarding the reliability of inclinometer measurements in children with CP has been found, however, there are many articles assessing its reliability in other fields of medicine and physiotherapy [35-38]. Although the inclinometer and goniometer reliability cannot be compared in this study because we did not measure hip abduction from the same hip position and with the same procedure, we would like to highlight the excellent inter-examiner reliability for inclinometer measurements. This may be because the inclinometer is very easy to use, since it is not necessary to make sure both the stationary and moveable extremities are aligned, as occurs with the goniometer. We also would like to mention that the reliability differences cannot be attributed completely to the instrument, as a different measurement protocol was used, and our current study design did not control for this possibility.

Both the goniometer and inclinometer have proved to be reliable in assessing hip movement [36]. Moreover, although there are some articles proving the reliability of goniometer measurements in children with CP, this is not true for measurements carried out with an inclinometer. Therefore, this pilot study attempted to establish the reliability obtained using an inclinometer in a sample of children with CP. However, more studies are required in this population group to further investigate the reliability of using an inclinometer to measure the mobility range of different joints, as well as comparing the goniometer and inclinometer measurements, although the inclinometer has some measurement limitations that make this difficult.

Few studies of inter-examiner reliability in children with CP have been carried out with more than three examiners; therefore, this should be taken into account for possible future studies. This factor is important, from our point of view, as children with CP are often assessed by a number of different health professionals and from multi-centre research where different examiners make measurements. At the very least, examiners carrying out these measurements and sharing the data should standardise their measuring method as far as possible. Since there is low inter-examiner reliability of goniometric measurements for CP patients, results from clinical examination should be treated with caution when making clinical decisions for CP patients.

As in other goniometric reliability measurement studies published, our main limitation was the small size of the sample [15,17-19,29], which was even smaller than in previous studies. However, this pilot study introduced novel aspects such as the analysis of the reliability of using an electronic inclinometer (so far, there has been no study in children with CP using this instrument), as well as the participation of five examiners for both goniometer and inclinometer studies.

## Conclusions

Inter-examiner reliability regarding the goniometric measurement of hip abduction in children with CP is low, in keeping with results found in previous publications. Therefore, goniometric measurements should be used cautiously when taking decisions, especially if no standardised protocols have been followed or no coordination/training session has been organised, as is the case for professionals who do not work within a team.

The inclinometer has proved to be a highly reliable instrument for measuring mobility in hip abduction in children with CP. This opens up new possibilities in this field, due to the greater reliability and user-friendliness of this instrument, which allow measurements to be taken very quickly. It cannot, however, measure all movements as the inclinometer uses the force of gravity when measuring, as the angle to be measured is perpendicular to the floor. Despite its limitations, new studies should be performed of its reliability in different joints and movements.

Further studies could be made on the reliability of the goniometer and inclinometer, as well as a comparison of the reliability of their measurements.

## List of abbreviations

CP: Cerebral palsy; ASIS: Anterior superior iliac spine; ICC: Intraclass Correlation Coefficient

## Competing interests

The authors declare that they have no competing interests.

## Authors' contributions

PH and EG were responsible for the concept of the project and the preparation of the manuscript.

PC was in charge of the sample and statistical analysis design.

The rest contributed to the description of the background, general design and definition of the different variables of study and their adaptation.

All authors have read, reviewed and approved the final manuscript.

## Pre-publication history

The pre-publication history for this paper can be accessed here:

http://www.biomedcentral.com/1471-2474/12/155/prepub
